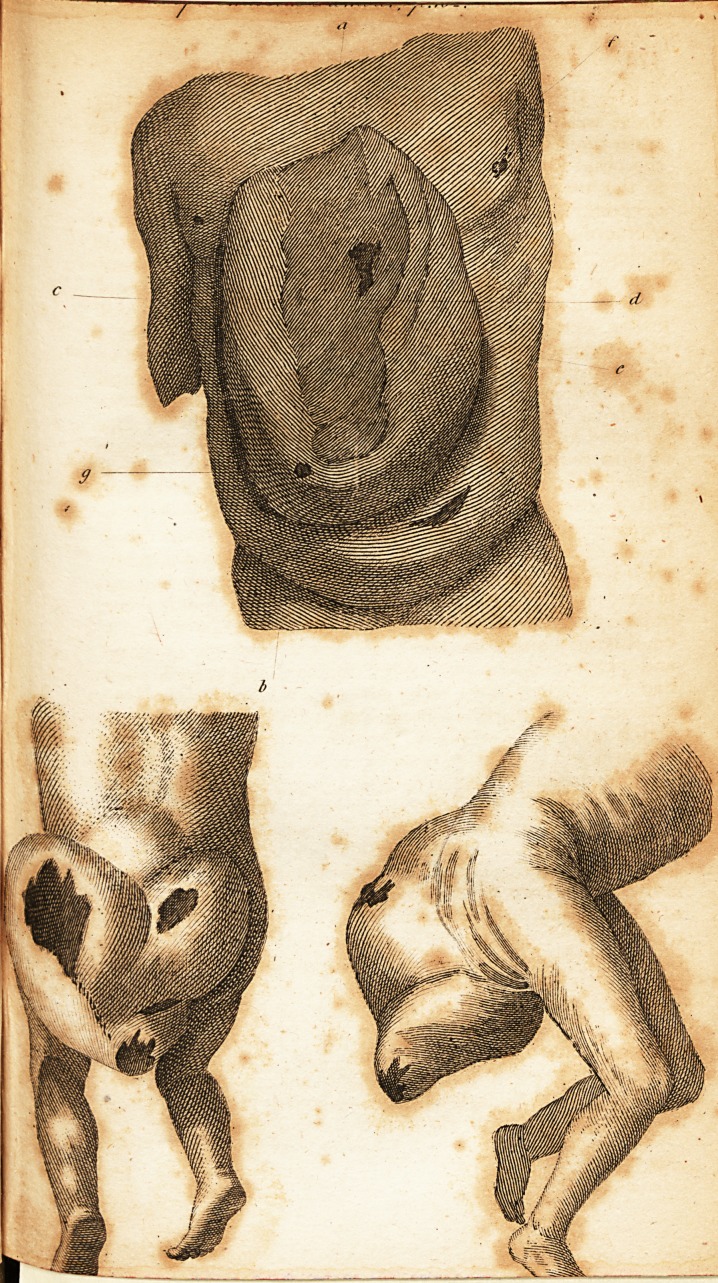# Mr. Leeson's Case of Semiosseous Tumour

**Published:** 1803-08-01

**Authors:** Beaumont Leeson

**Affiliations:** Grantham


### *To the Editors of the Medical and Physical Journal.*

Gentlemen,

I Have enclosed the description of a Case, which, although it may suggest no improvement in practice, may, from its curiosity, merit a place in your useful publication.

Francis Otter, by a fall in 1796, bruised his side, and was supposed to break one or more of his ribs; but no particular inquiry was then made as to the nature of the accident, nor any medical assistance required. From this time he perceived a small tumour in the centre of the first false rib, which continued gradually to increase, without occasioning any extraordinary pain or inconvenience, until it attained the very uncommon dimensions described in the annexed drawing; by which it is seen to occupy the whole of the right hypochondric, right iliac, and epigastric regions; to extend upwards beneath the sternum, and downwards near to the umbilicus: the circumference at the base being two feet ten inches and a half; the broadest diameter, as shewn by the line A.B. one foot and one quarter of an inch; the narrowest diameter, according to the line C. D. eight

D. eight inches and three-quarters. The line E. is intended to mark the height of the tumour, above the parietes of the abdomen, from which it was gradually elevated to the extremity of that line. The external surface exhibited no remarkable appearance, being covered with enlarged veins, until a few days previous to the breaking of the tumour, when the more elevated portion became of a livid hue, and the part F. put on the appearance of incipient gangrene. On examination, it was very evident that the marginal parts of this tumour were formed of bone, and the centre likewise gave a sensation of resistance greater than cartilage, but less than bone. On the upper part it was easy to remark, the sternum forming that boundary of the tumour thrown upwards from its situation. I first saw this case in 1798, and from that time had marked its progress with considerable attention, being by no means satisfied with the opinion I had formed concerning it. I had never failed to advise the man to use no other means than such as were necessary to guard the surface from friction, and by no means to suffer the part to be opened either by the knife or by caustic, a plan which had been proposed by some empiric. The patient was the more inclined to attend to this instruction, as he suffered no other inconvenience, than such as arose from the size of the tumour; being able to pursue his daily labour, which was that of a labourer on the road, until about three months ago; to lie down in bed and sleep undisturbed; to eat heartily, and with appetite. Latterly, the size of the tumour has rendered him very unweildy, and this, together with his age, which is now 80, has prevented him continuing his occupation. I supposed the tumour to arise from aneurism of one of the intercostal arteries; and having explained the nature and danger of the disease, had charged the patient to inform me immediately on the rupture of it.

On the 5th of June, 1803, I was informed this event had taken place; and on visiting the patient, I found a small opening, nearly in the centre, marked by the letter F, from which a large quantity of a fluid, resembling pus, tinctured with blood, was poured out; it was glairy, and perfectly inodorous; the quantity discharged could not be less than a gallon in the twenty-four hours, that which was preserved filling two large sized chamber pots.

On the 6th, the discharge continued, pulse intermitting, extremities cold, complaining of a little pain on pressing the lower part of the abdomen.

 7th.

7th, The discharge much abated; pulse firm and regular; has eaten with appetite.

8th, The discharge has ceased, leaving the tumour flaccid, with a gangrenous slough about to separate around the orifice; pulse firm and good.

9th, The discharge has recommenced; the pulse good, but pain in the lower part of the tumour very great; tongue dry.

10th, Has passed a quiet night, but is this morning very restless; pulse intermits every fourth pulsation; the tongue moist; the gangrenous disposition of the skin more extended; the discharge very profuse, and most uncommonly offensive.

11th, From the state of the patient yesterday, I concluded he could not long continue; and was much surprised to find this morning, that the discharge was much abated in quantity, and much less offensive; the pulse was good and regular, and nourishment had been taken in abundance, with appetite; the tumour was much contracted.

13th. The tumour continues to discharge, and to settle; pulse is firm and regular; the entrance of the abscess presents a projecting substance, which when drawn out proves to be a portion of the rib in a state between bone and cartilage; much more of the same substance was also removed, and a considerable quantity of offensive matter pressed out.

14th, The pulse was very regular and firm, the tongue moist, the tumour much diminished but bulging very considerably at the lower part. In order to evacuate the matter more readily, I made an opening at the lower part, marked G; some florid blood flowed out, but the wound was soon plugged up by the semi-osseous substance contained in the tumour. At first the patient complained of violent pain; the pulse was interrupted, and the whole frame appeared to suffer a considerable shock. In the evening, the lower orifice had discharged the same fetid matter mixed with semi-osseous substance, which had protruded from the upper orifice; pulse regular, appetite good. From this time to the present day, the patient has continued daily to amend; for some time, cartilaginous substance was discharged with very offensive matter; this has gradually diminished; and now the discharge is a laudable and good pus in moderate quantity; some exfoliations have already taken place, and more may be expected; the tumour is so much contracted, that there is not more space than two inches between the superior and inferior orifice. The internal ternal surface of the abscess exhibits healthy granulations; every present appearance makes the recovery of the patient undoubted.

I cannot omit this opportunity of returning my public thanks to Mr. Hartley, to whom I am obliged for the very accurate sketch which accompanies this description, by which the situation and extent of this extraordinary disease is rendered much more evident than words alone could make them.

Having given as accurate an history of the progress of this disease, as my opportunity of acquiring information would admit, I shall defer to a future number, some observations on the nature of the disease; and its application to illustrate some of the laws of the animal œconomy.

I am, &c.

*Grantham,*

*July* 3, 1803. BEAUMONT LEESON, jun.

DESCRIPTION OF THE PLATE.

A. B. The longest diameter of the tumour.
C. D. The shortest diameter.
E. A line intended to mark the elevation of the tumour above the abdomen.
F. The opening formed by mortification.
G. The artificial opening made by incision.

## Figures and Tables

**Figure f1:**